# Lower Muscle and Blood Lactate Accumulation in Sickle Cell Trait Carriers in Response to Short High-Intensity Exercise

**DOI:** 10.3390/nu14030501

**Published:** 2022-01-24

**Authors:** Laurent A. Messonnier, Samuel Oyono-Enguéllé, Lucile Vincent, Hervé Dubouchaud, Benjamin Chatel, Hervé Sanchez, Alexandra Malgoyre, Cyril Martin, Frédéric Galactéros, Pablo Bartolucci, Patrice Thiriet, Léonard Féasson

**Affiliations:** 1 Université Savoie Mont Blanc, Laboratoire Interuniversitaire de Biologie de la Motricité, EA 7424, F-73000 Chambéry, France; lucile74.xxc@gmail.com (L.V.); benjamin.chatel@live.fr (B.C.); 2 Université de Yaoundé 1, Laboratoire de Physiologie, Faculté de Médecine et des Sciences Biomédicales, Yaoundé, Cameroon; hubert.freund@wanadoo.fr; 3 Université Grenoble Alpes, Inserm, LBFA, F-38000 Grenoble, France; herve.dubouchaud@univ-grenoble-alpes.fr; 4 Institut de Recherche Biomédicale des Armées, Unité Physiologie de l’Exercice et des Activités en Conditions Extrêmes, F-91223 Brétigny-sur-Orge, France; hsanchez@mtraining.fr (H.S.); alexandra.malgoyre@intradef.gouv.fr (A.M.); 5 Université de Lyon, Université Claude Bernard Lyon 1, Laboratoire Interuniversitaire de Biologie de la Motricité, EA 7424, F-69100 Villeurbanne, France; cyril.martin@univ-lyon1.fr (C.M.); patrice.thiriet@univ-lyon1.fr (P.T.); 6 Sickle Cell Referral Center, Department of Internal Medicine, Henri-Mondor University Hospital-UPEC, AP-HP, F-94000 Créteil, France; frederic.galacteros@aphp.fr (F.G.); pablo.bartolucci@aphp.fr (P.B.); 7 Université de Lyon, Université Jean Monnet Saint-Etienne, Laboratoire Interuniversitaire de Biologie de la Motricité, EA 7424, F-42000 Saint-Etienne, France; leonard.feasson@univ-st-etienne.fr; 8 Referent Center of Neuromuscular Diseases Euro-NmD, Myology Unit, Department of Clinical and Exercise Physiology, University Hospital of Saint-Etienne, F-42055 Saint-Etienne, France

**Keywords:** lactate transport, pH regulation, gluconeogenesis, recovery

## Abstract

It remains unclear whether sickle cell trait (SCT) should be considered a risk factor during intense physical activity. By triggering the polymerization-sickling-vaso-occlusion cascade, lactate accumulation-associated acidosis in response to high-intensity exercise is believed to be one of the causes of complications. However, our understanding of lactate metabolism in response to high-intensity exercise in SCT carriers is incomplete. Thirty male SCT carriers (*n* = 15) and healthy subjects (*n* = 15) with and without α-thalassemia performed a 2-min high-intensity exercise. Blood and muscle lactate concentrations were measured at exercise completion. Time courses of blood lactate and glucose concentrations were followed during the subsequent recovery. Additional biochemical analyses were performed on biopsies of the *vastus lateralis* muscle. SCT was associated with lower blood and muscle lactate concentrations in response to the short high-intensity exercise. Compared to controls, the muscle content among SCT carriers of lactate transporter MCT4 and β_2_-adrenergic receptor were higher and lower, respectively. During recovery, the lactate removal ability was higher in SCT carriers. In the present study, no effect of α-thalassemia was observed. The lower blood and muscle lactate accumulations in SCT carriers may, to some extent, act as protective mechanisms: (i) against exercise-related acidosis and subsequent sickling, that may explain the relatively rare complications observed in exercising SCT carriers; and (ii) against the deleterious effects of intracellular lactate and associated acidosis on muscle function, that might explain the elevated presence of SCT carriers among the best sprinters.

## 1. Introduction

Disagreement exists about whether sickle cell trait (SCT) carriage should be considered a benign condition or a risk factor during intense physical activity [1,2,3,4,5,6]. Considering SCT a risk factor is based on the fact that during high-intensity exercise, SCT carriers are prone to hemorheological disturbances (augmented blood viscosity, decreased red blood cell deformability and increased endothelial activation), intravascular coagulation and sickling [7,8,9,10,11]. SCT as a risk factor becomes particularly important when intense exercise is performed at altitude, and is associated with dehydration or hyperthermia. In such circumstances, serious complications (such as heat stroke, hyperkalemia, hematuria, pulmonary edema, cardiac arrhythmias and ischemia, splenic infarction, renal failure, myalgias, vaso-occlusion crisis (VOC) and fulminant rhabdomyolysis) have been reported, which can ultimately lead to sudden death [3,5,12,13,14,15,16]. Moreover, the occurrence of complications, collapse and sudden death during the first 30–60 min following high-intensity exercise^14^ demonstrates the need to pay particular attention to this apparently critical postexercise period in SCT carriers.

High-intensity exercise induces significant activation of glycogenolysis and glycolysis, leading to muscle lactate production and accumulation, and subsequently, to increased blood concentrations. High elevations of blood lactate concentrations are accompanied by systemic acidosis [17], which is often seen as the likely triggering factor of accidents in SCT carriers [13]. The main reason for this is that acidosis decreases, by the Bohr effect, the affinity of hemoglobin (Hb) for oxygen, thus releasing it early in the microcirculation so that HbS polymerization, and consequently sickling, is favored [18].

The persistence of risk of VOC and complications several minutes after exercise [14] lies in the combination of two particularities of the postexercise blood lactate kinetics. First, lactate release by muscles continues for several minutes after exercise completion [19], leading to a postexercise increase of blood lactate concentrations during this period [20]. Second, blood lactate concentrations remain elevated for 30–45 min after intense exercise, disturbing the acid/base balance for a long period postexercise [20] that may potentially favor the occurrence of VOC and accidents several minutes after intense exercise. 

The lower index of oxygen supply to tissues and muscle oxidative potential (e.g., cytochrome *c* oxidase activity) [8,21] provide support for the hypothesis of a higher nonoxidative glycolytic energy supply, and consequently, higher blood lactate accumulation in response to maximal exercise in SCT [20], giving carriers a possibly augmented risk of exercise-related complications. However, our knowledge of lactate metabolism in the context of SCT is relatively sparse (restricted to blood data) and contradictory [20,22,23,24,25,26,27,28,29]. For instance, Freund et al. [20] found higher, Gozal et al. [22], Bilé et al. [24] and Sara et al. [25] found lower, and Marlin et al. [26,30] found similar blood lactate concentrations in SCT carriers compared to healthy controls in response to incremental exercises up to exhaustion. Bilé et al. [27] also found similar blood lactate concentrations in SCT carriers and control counterparts during and after repetition of 6-s maximal exercise bouts on a cycle ergometer. The conflicting results between these studies might be related to the low number of subjects studied (*n* ≤ 9) [20,22,24,25,26,27,30], differences in the types of exercise performed [27], different environments [23] and different physical ability between the studied populations [20]. Moreover, because of silent/subclinical repercussions of SCT [8,21,31], one cannot exclude that the same physical activity (which is kind of a training load) results in divergent physical ability in SCT carriers and healthy subjects. In that context, it may also be important to take into account the daily physical activity among the studied populations [21,31]. Complementary studies on lactate metabolism in exercising SCT carriers are thus warranted.

Thalassemia constitute another type of genetic mutation frequently observed in African origin populations. SCT is singularly associated with α-thalassemia (α-t). Subjects with the dual hemoglobinopathy (SCTα-t) often display lower HbS percentage and microcytosis [32,33]. In several respects, these particularities mitigate pathophysiology of sickle cell disease by favoring the flow of red blood cells through the capillaries, and by dampening the risk of sickling [32,34]. From that point of view, α-thalassemia deserves to be considered. 

The present study aimed to assess parameters of lactate metabolism in carriers of SCT in response to short, high-intensity exercise, and parameters of lactate time-course during the subsequent recovery. The studied parameters refer mainly (i) to lactate accumulation in different compartments (muscle and blood), (ii) to sarcolemmal lactate transport and its regulatory mechanisms, and (iii) to lactate removal ability. 

## 2. Methods

### 2.1. Subjects 

Thirty adult Cameroonian male SCT carriers (SCT, *n* = 15) and healthy subjects (CON, *n* = 15), with or without α-thalassemia, volunteered to participate in the study. They were assigned to one of the four groups: CON (*n* = 10), CONα-t (*n* = 5), SCT (*n* = 6) and SCTα-t (*n* = 9). Recruitment was conducted by posted notices and word of mouth in the students’ community of the University of Yaoundé. Age, height and weight were 24 ± 2 years, 173 ± 5 cm, and 67 ± 5 kg (means ± standard deviation). The study was conducted at the General Hospital of Yaoundé in Cameroon. The study conformed to the guidelines set by the Declaration of Helsinki for human studies and was approved by the local ethics committee (no. 10-12-2005). Before giving their written informed consent, all subjects were fully informed of the objectives and possible risks and discomforts due to the experiments. 

Volunteers who (a) presented a hemoglobinopathy other than SCT and α-thalassemia, (b) had suffered from a malaria episode within the previous 2 months, (c) were taking any medications, (d) were HIV carriers, (e) were taking part in another research program and/or (f) were smokers and/or regular alcohol drinkers, were not included in the study. At inclusion, blood samples were drawn from the antecubital vein of the nondominant arm at rest, and blood samples were then assayed for Hb and α-thalassemia. 

### 2.2. Experimental Design

The protocol consisted of three visits (V1–V3) which took place a week apart. 

#### 2.2.1. Inclusion (V1) 

All prospective subjects underwent a thorough physical examination, anthropometric measurements and blood sampling. Blood samples were drawn from the antecubital vein of the nondominant arm at rest and assayed for Hb. Positive test results for SCT were determined by the presence of HbS at a level lower than 50% of total hemoglobin using HPLC. The presence of α-t was detected with a single-tube, multiplex-PCR assay, capable of detecting any combination of the six common single and double gene deletions in α-t. Only one form of α-t was found in the present study, the heterozygous form marked by the deletion of 3.7 kb of DNA containing one of the two linked α-globin genes (αα/-α [3,7]). Subjects also performed preliminary incremental and maximal exercise bouts to become familiar with the procedures.

#### 2.2.2. Incremental Exercise Test to Exhaustion (V2) 

Subjects performed a graded exercise test using a leg-cycle ergometer (Kettler, Ense-Parsit, Germany). The exercise stopped at volitional exhaustion i.e., when the subjects were no longer able to sustain the work rate at the required pedaling frequency of 70 rpm. The exercise started at 70 W. After 3 min of exercise at this load, the work rate increased by 35 W every 3 min until volitional exhaustion. Heart rate (HR, beats·min^−1^) was measured continuously using a chest belt (Polar Electro, Kempele, Finland). This exercise session was used for determination of maximal heart rate (HR_max_, beats·min^−1^) and the work rate associated with HR_max_ (P_max_, W and W·kg^−1^). 

#### 2.2.3. Short High-Intensity Exercise Bout (V3) 

For the short high-intensity exercise, subjects were requested to lie down on a bed in the dorsal decubitus position. Then, the right leg was prepared for muscle biopsy. After shaving, asepsis was obtained using alcohol and iodized derivatives, and a local anesthesia of cutaneous and subcutaneous tissues was made (2% lidocaine), without crossing the muscular aponeurosis. An incision not exceeding 8 mm broad was made (at a level corresponding to one-third of the distance from the upper margin of the patella to the anterior superior iliac spin) until the crossing of the epimysium. A temporary bandage protected the incision until subsequent (post exercise) muscle biopsy (*vide infra*). After 30 min of rest, a hyperemic cream (Dolpyc^®^, Pfizer, New York, NY, USA) was applied to the left earlobe for postexercise blood micropunctures. Then, the volunteers performed a 10-min warm-up at a heart rate of 130 beats·min^−1^ (~50% of P_max_). After a 5-min rest, a blood micropuncture (20 µL) was collected from the right earlobe (pre-exercise value) and the hyperemic cream was removed from the left earlobe. Then, the subjects cycled for 2 min at 110% of P_max_. Immediately at exercise completion, the muscle biopsy was rapidly performed and immediately immersed in liquid nitrogen. The delay between exercise completion and freezing averaged 10 s. Hemostasis was then ensured by a 5-min compression, and the access closed by sterile strips. The biopsy was used for determination of (i) postexercise muscle metabolite concentration and (ii) muscle content of proteins of interest (*vide infra*). Blood micropunctures (20 µL) were also collected from the left earlobe at exercise completion, and thereafter at 0.5, 1, 1.5, 2, 2.5, 3, 3.5, 4, 4.5, 5, 6, 8, 10, 12, 15, 20, 25, 30, 40, 50, 60, 70 and 80 min of passive recovery. Arterialized capillary blood sampled at the earlobe was diluted in a hemolyzing solution and stored at 4 °C until analysis. Blood samples were used to determine the time-course of lactate and glucose concentrations during recovery. 

### 2.3. Blood Lactate and Glucose Concentrations and Their Time-Courses during Recovery 

Lactate and glucose concentrations were determined enzymatically in whole blood using a YSI 2300 analyzer (YSI Inc., Yellow Springs, OH, USA). 

Individual blood lactate recovery curves were fitted to the biexponential time function [35].

La(t) = [lactate]_b_(0) + A_1_(1-e^−^^γ^^1·t^) + A_2_(1-e^−^^γ^^2·t^) (1)

where [lactate]_b_(0) and La(t) (mmol·L^−1^) are blood lactate concentrations at exercise completion and at a given time during recovery, respectively. Concentration parameters A_1_ and A_2_ (mmol·L^−1^) are the amplitudes of the exponential functions. The velocity constants γ_1_ and γ_2_ (min^−1^) represent the ability to exchange lactate between the previously active muscles and the blood and the overall ability to remove lactate, respectively [36]. The blood lactate recovery curves were fitted to Equation (1) by iterative nonlinear regression using KaleidaGraph 4.0 software (Synergy Software, Reading, PA, USA) to determine the values of [lactate]_b_(0), A_1_, γ_1_, A_2_, and γ_2_. 

### 2.4. Muscle Analyses

Concerning the biochemical analyses, a first part of the postexercise muscle samples was freeze dried (Lyovac GT2, Leybol-Heraeus, Köln, Germany), dissected free from connective and fatty tissue and blood, and powdered in a chamber of controlled humidity (<40% relative humidity). For metabolites concentration determination, muscle powder was extracted with HClO_4_ (650 mmol·L^−1^), neutralized, and assayed enzymatically by fluorometric analysis. Metabolite concentrations are expressed in millimoles per kilogram tissue dry mass (mmol·kg^−1^). For LDH isoforms determination, samples were placed into an ice-cold homogenization buffer (30 mg wet weight/mL) containing: 5 mM Hepes (pH 8.7), 1 mM EGTA, 1 mM dithiothreitol, 5 Mm MgCl2, and 0.1% Triton. Samples were homogenized using a micro-glass hand homogenizer and were incubated for 60 min at 0 °C to ensure complete enzyme extraction. The LDH isoenzyme profile was determined using agarose gel electrophoresis (Sigma LDH reagent kit, Sigma) at 200 V for 90 min. Isoenzyme bands were visualized and quantified using an image analysis system (Bio-Rad). 

The second part of the biopsy was used for the quantification of selected proteins as previously described [37]. Approximately 30 mg of muscle were homogenized (Polytron 2100, Kinematica, Newark, NJ, USA) in a sucrose buffer (250 mM sucrose, 30 mM HEPES, 2 mM EGTA, 40 mM NaCl, 2 mM PMSF, pH 7.4) and centrifuged at 1000× *g* for 5 min. This procedure removed heavy material, including a fraction of the mitochondria. The supernatant was spun at 190,000× *g* for 90 min at 4 °C. The new supernatant (cytosolic fraction) was stored at −80 °C, while the new pellet (total muscle membrane fraction, including sarcolemmal and mitochondrial membrane fractions) was resuspended in Tris-SDS (10 mM Tris, 4% SDS, 1 mM EDTA, 2 mM PMSF, pH 7.4). Protein content was determined with a BSA standard (DC protein assay, Bio-Rad, Herlev, Denmark). Ten micrograms of protein from each sample were subjected to SDS-PAGE (excel 8–18% gradient gel; Amersham Biosciences, Uppsala, Sweden) and electroblotted to a Immobilon-P transfer membrane (Millipore, Copenhagen, Denmark). A Ponceau staining allowed us to check the homogeneity of load between wells. The membrane was then blocked with a buffer containing 1% BSA, 0.1% Tween 20, and 0.5% low fat milk and further incubated with the primary antibody diluted in the same blocking buffer. After treatment with the secondary antibody and repeated washing, the membrane was incubated with enhanced chemiluminescence reagent (Amersham Biosciences) and visualized on a hyper film (Amersham Biosciences). Quantification of the selected protein was performed by scanning the film and analyzing band densities with the SigmaGel software (SPSS, Chicago, IL, USA). The membrane-bound lactate/H^+^ cotransporter MCT1 and MCT4 (both 43 kDa) were measured on the muscle membrane fraction, whereas the CAII and CAIII (both 31 kDa) were measured on the cytosolic fraction. The antibodies for NHE1 (no. MAB3140), MCT1 (no. AB3540P), MCT4 (no. AB3316P), and CAII (no. AB1828) were purchased from Chemicon (Chandlers Ford, UK), from Spectral Diagnostics (Toronto, Canada) for CAIII (no. 4020), and from Santa Cruz Biotechnology INC (Dallas, TX, USA) for β2 AR (sc-569). Some membranes were reused after treatment with a stripping solution (Re-Blot Plus, Chemicon). 

### 2.5. Statistical Analysis 

Normality of distribution was tested by a Shapiro-Wilk test. Descriptive statistics are expressed as means (standard deviation) or as median (minimum-maximum). A two-way ANOVA was used to determine the effects of HbS, α-thalassemia and their interaction on the different measured parameters (StatView, SAS Institute, Cary, NC, USA). Excepted some hematological data, no effects of α-t and interactions SCT:α-t were observed on the variables of interest (Supplemental Tables S1–S3). Therefore, subjects with and without α-t were pooled in the SCT and CON groups. T-test and Mann-Whitney test were used to determine the effect of HbS on the different measured parameters (JASP 0.11.1, https://jasp-stats.org/, accessed on 30 October 2021). Differences between values were considered to be significant for *p* ≤ 0.05. 

## 3. Results 

The anthropometric, physiological and hematological characteristics of the subjects are reported in Table 1 [21]. The two groups of subjects were matched in terms of age, body mass, maximal heart rate, exercise capacity (assessed by P_max_) and daily energy expenditure, suggesting that the groups were homogenous regarding physical characteristics and fitness. In addition, MCV and MCH were lower in SCT likely due to the higher proportion of α-t in this group. None of the subjects experienced adverse events during or after either the incremental or short supramaximal exercise bouts. 

### 3.1. Blood Lactate Response to Short Supramaximal Exercise

At rest, blood lactate concentrations were not different between groups (Table 2). In response to the short supramaximal exercise bout, blood lactate concentrations increased drastically, but to a lesser extent in SCT carriers (Table 2). 

### 3.2. Muscle pH Regulation, Lactate Transport, Metabolite Concentrations and Isoforms of Lactate Dehydrogenase 

The muscle content of carbonic anhydrase (CA) II and III, sodium-bicarbonate cotransporter (NBC) and monocarboxylate (MCT) 1 was not different between groups. On the other hand, the muscle content of MCT4 was higher, and that of β_2_-adrenergic receptor was lower in SCT compared to controls (Table 2). Furthermore, muscle lactate concentrations were significantly lower in carriers of SCT in response to the short supramaximal exercise (Table 2). Concentrations of other muscle metabolites did not diverge between groups (Table 2). Proportions of M and H forms of lactate dehydrogenase (M-LDH and H-LDH, respectively) were not different between groups (Table 2). 

### 3.3. Blood Lactate and Glucose Kinetics during Recovery

**Figure 1:**
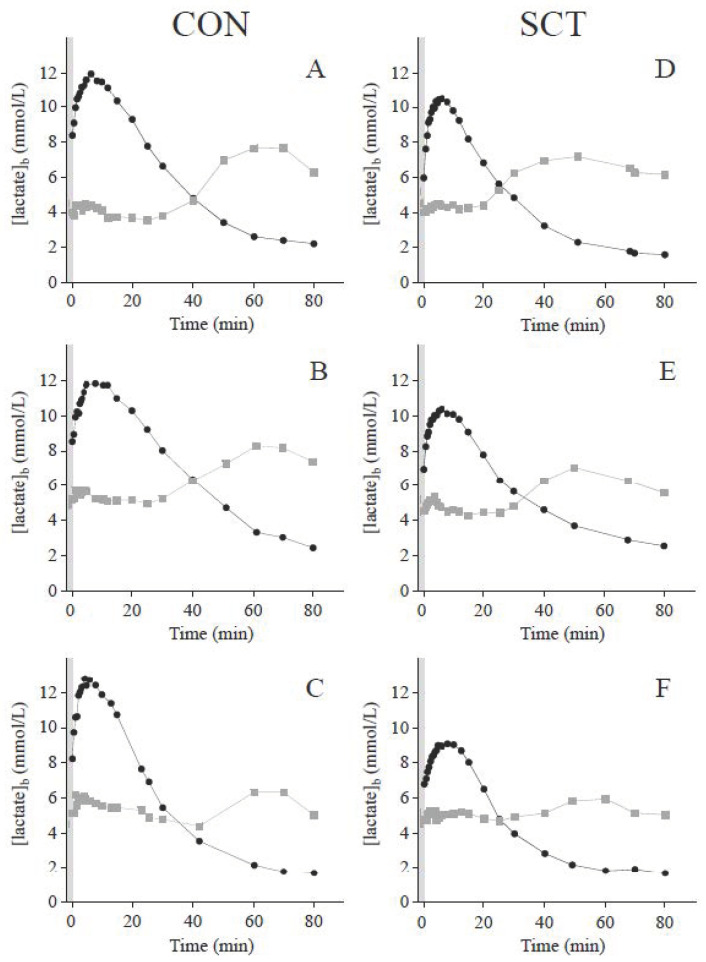
Typical recovery time-courses of blood lactate (black circles) and glucose (grey squares) concentrations obtained in control subjects (panels **A**–**C**) and SCT carriers (panels **D**–**F**).

Time courses of blood lactate concentrations during recovery displayed typical bi-exponential shapes (Figure 1) [38]. γ_1_, which assesses the lactate exchange ability between the previously active muscles and the blood, was not different between groups. On the other hand, the lactate removal ability was significantly higher in the SCT carriers, as demonstrated by their higher γ_2_ values compared to their control counterparts (Table 3).

As recently depicted, a rebound of glycemia was observed after 20–50 min of recovery [38]. The cross-over point between the glycemia and blood lactate concentration curves took place at the same concentrations among groups but occurred earlier in the SCT groups compared to the control groups (Table 3).

## 4. Discussion

The aim of the present study was to improve our knowledge of lactate metabolism in response to high-intensity exercise in SCT carriers. The main findings were that, compared to control subjects, SCT carriers exhibited (i) lower muscle and blood lactate accumulations in response to short high-intensity exercise, (ii) a higher muscle content of MCT4 and (iii) a more rapid decrease of blood lactate levels during the subsequent recovery. 

### 4.1. Metabolic Response to Short Supramaximal Exercise in SCT Carriers

In the present study, we found lower blood lactate accumulation in response to short high-intensity exercise in SCT carriers. This observation, although surprising in the light of the literature suggesting a higher contribution of the nonoxidative glycolytic pathway in energy contribution [8,21], is actually reminiscent of previous studies [22,23,24]. Because capillary density, which determines lactate release from muscles [39], was lower in SCT carriers than controls in the present study [21], we initially hypothesized (H1) that the lower blood lactate concentrations in response to short high-intensity exercise might have been due to a slower lactate release from the active muscles. We also hypothesized (H2) that the resulting retention of lactate and H^+^ in muscle may augment the acid/base balance disturbances and induce a long-term responsive upregulation of muscle buffering mechanisms in SCT carriers. Concerning H2, no divergences were noticed between groups concerning the muscle content of CAII, CAIII and NBC, suggesting the lack of particular adaptations in the bicarbonate-related mechanisms of muscle pH regulation with SCT. Along the same line, the muscle content of MCT1, which cotransport lactate and H^+^ in a 1:1 ratio and thus may participate in the muscle pH regulation [40] was not different between groups. The lack of difference in MCT1 between groups is not so surprising inasmuch as MCT1 is mainly involved in lactate and H^+^ uptake by myocytes [41]. On the other hand, interesting results were observed on the muscle content of MCT4 which was significantly higher with SCT in the present study. MCT4 is particularly involved in lactate and H^+^ extrusion from the myocytes [42] and is therefore a strong contributor to pH regulation during high-intensity exercise [43]. 

By essence (lactate-H^+^ coupled-transport), MCT4 is also the main pathway by which lactate is extruded from the active muscles [42]. From that point of view, the higher muscle MCT4 content may counterbalance the lower density of the muscle capillary network [21] to maintain an adequate lactate release from the muscles. In accordance with this line of reasoning, but contrary to our initial hypothesis (H1), the lactate exchange ability between muscle and blood (assessed by γ_1_) was not different among groups and therefore cannot explain the lower blood lactate accumulation. Other mechanisms should be involved. A previous study reported that MCT4, was upregulated by hypoxia [44]. Whether the higher muscle MCT4 content we observed in the present study in SCT carriers is related to (i) hypoxic episodes, (ii) an adaptive response to regular muscular acid/base balance disturbances, (iii) a compensatory mechanism of lower capillary density, or (iv) a combination of the previous possibilities, remains to be determined. 

Because the lactate exchange ability cannot be incriminated to explain the lower postexercise blood lactate concentrations, then lower muscle lactate accumulation must be considered. This idea may appear surprising given the lower index of oxygen supply to tissues and the depressed muscle oxidative potential reported in SCT carriers [8,21] However, significantly lower muscle lactate concentrations were measured in the SCT carriers than in the control subjects. The lower muscle lactate accumulation in SCT carriers cannot be attributed to a default in muscle glycolytic potential since the key enzymes of glycolysis [31] and the M-LDH proportion (Table 2), which all drive lactate production, were similar among groups. Consequently, a lower activation of glycogenolysis and/or glycolysis should be considered. If a lower activation occurred, it could not be mediated by changes in the ATP/ADP ratio since this ratio was not different among groups (Table 2). On the other hand, lactate production is known to be intimately related to muscle glycolytic pathway activation by catecholamines [45]. Interestingly, the sarcolemmal content of β_2_-adrenergic receptors was depressed in the SCT groups, that might to some extent support the idea of lower activation of glycogenolysis and glycolysis in the carriers and their lower muscle lactate accumulation. Other possibilities may exist. For example, a higher lactate removal ability during exercise could be evoked and in view of the results obtained during recovery (*vide infra*), one cannot totally exclude this possibility. As a whole, further studies are necessary to understand the intriguing lactate metabolism depicted in SCT carriers. 

### 4.2. Postexercise Blood Lactate Kinetics and Its Relation with Glycemia 

During recovery, blood lactate concentrations remained elevated (>5 mmol·L^−1^) for 20–50 min (Figure 1) [38]. Interestingly, blood lactate levels during this period were lower and decreased more rapidly in the SCT carriers than in their control counterparts (Table 3). This can be attributed to the higher lactate removal ability during recovery (assessed by γ_2_) observed in SCT carriers (Table 3). This latter result reinforces the possibility of a higher lactate removal during exercise (*vide supra*) in SCT carriers. 

Correlated with the muscle oxidative capacity (citrate synthase activity and mitochondrial respiration) and content of MCT1 (involved in lactate uptake by the muscle) [46,47], γ_2_ is believed to be mainly attributed to oxidation of lactate during recovery. From that point of view, the higher γ_2_ values of SCT carriers suggest a higher lactate oxidation during recovery in these subjects. The second main fate of lactate during recovery is hepatic gluconeogenesis [48]. The quicker lactate disappearance rate constant in SCT carriers may also indicate an earlier lactate uptake by the liver [48]. This hypothesis is supported by the fact that the delayed postexercise rebound of glycemia attributed to glucose and lactate interactions (via gluconeogenesis) [38] was observed earlier postexercise in SCT carriers (Table 3). Lactate uptake by the liver is intimately related to lactate delivery, and thus to local blood flow. Interestingly, local blood flow is driven by the sympathetic nervous system activation and especially its hormones: the catecholamines. If, as already suspected during exercise (*vide supra*), catecholamines action is mitigated, this would limit vasoconstriction at the splanchnic level, maintain hepatic local blood flow, preserve lactate delivery to the liver, and ultimately favor lactate uptake. 

### 4.3. Effects of α-Thalassemia 

In the present study, α-thalassemia did not induce any specific muscle metabolic responses/adaptations to exercise. This is reminiscent with the lack of effects of α-thalassemia on muscle structural and energetic characteristics previously reported in SCT and CON [31]. At the vascular level, α-thalassemia seems (i) to improve RBC deformability and blood apparent viscosity, (ii) to blunt inflammation and adhesion and (iii) to dampen microvascular remodeling [9,10,21]. Taken together, the present results and the literature seem to indicate that skeletal muscle (at least its structural and energetic characteristics as well as its metabolic responses to exercise) does not seem to take advantage of α-thalassemia whom effects at the vascular level seem to be tangible [9,10,21].

### 4.4. Clinical Relevance and Consequences on High-Intensity Exercise Performance 

The lower index of oxygen supply to tissues and the depressed muscle oxidative potential [8,21] were evidence of a higher nonoxidative glycolytic energy supply, and consequently, of higher blood lactate accumulation in response to short high-intensity exercise in SCT [20]. However, contrary to this idea, we clearly demonstrated lower muscle and blood lactate accumulations delineating kind of a paradox in exercising SCT carriers.

The lower blood lactate accumulation may constitute a protective mechanism to limit blood acidification during and after exercise that may also contribute to restrict the risk of sickling and complications in SCT carriers. This protective mechanism may at least partly explain, despite substantial literature reporting sudden deaths in carriers of SCT, why these fatal episodes remain rare in respect to the prevalence of SCT in the considered populations [14]. Our results may also enlighten why severe complications have been observed almost exclusively after extreme exertional exercise which (i) may overwhelm this protective mechanism, and (ii) are often associated with dehydration and hypoxia that also worsen the risk of sickling and thus of VOC. Our results may also support the results of Nelson et al., who found no significant difference in the risk of death among soldiers with and without the sickle cell trait, although the authors noted a higher risk of exertional rhabdomyolysis in SCT carriers [49]. 

Moreover, the lower muscle lactate accumulation in response to high-intensity exercise may also partly explain why the prevalence of the trait is higher in the best athletes involved in short high-intensity exercise, e.g., short sprints [50,51]. Indeed, if during moderate-intensity exercise lactate can be used as an important energy substrate for muscle contraction [45], significant lactate accumulation and the accompanying acidosis may, on the contrary, lead to alterations of muscle function [52,53,54,55,56,57,58,59], although this view has been challenged [60,61]. Nevertheless, following this line of reasoning, the lower lactate accumulation in SCT carriers might account for better maintenance of muscle function during exercise and thus explain the better performance during high-intensity exercise in SCT carriers [50,51].

### 4.5. Nutritional and Metabolic Flexibility Perspectives/Hypotheses

During exercise, the use of a lactate clamp has been shown to increase lactate disposal [45] and reduce glucose utilization [62]. Furthermore, lactate-supplemented sport drinks have been shown to increase lactate disposal and to improve performance [63]. If the higher lactate removal ability is confirmed in SCT, these previous studies suggest that carriers may take more advantage than controls from lactate clamp or supplementation for lactate utilization and physical performance. Besides, because blood lactate accumulation limits lipolysis and free fatty acids oxidation [64], the greater lactate removal ability of SCT carriers may, by lowering blood lactate levels, provide them a different substrate partitioning and/or metabolic flexibility. Further studies are necessary to confirm these possibilities/hypotheses. 

### 4.6. Limitations and Perspectives

The main limitation of the present study is the low number of subjects in each group. This limitation may account for the lack of effects of α-thalassemia found at the muscular level [31]. Further studies involving a higher number of subjects are thus necessary to confirm or refute the lack of role of α-thalassemia at the muscular level. The present results should also be complemented with tracer studies. Such studies would make it possible to determine the effects of SCT on important parameters of lactate kinetics (e.g., lactate rates of appearance, disappearance and oxidation, as well as the metabolic clearance rate of lactate).

## 5. Conclusions

The main findings of the present study are that carriers of sickle cell trait displayed (i) lower muscle and blood lactate accumulations in response to a short high-intensity exercise bout, (ii) higher muscle MCT4 content and (iii) more rapid decrease of blood lactate concentrations during recovery, than control counterparts. As a whole, the lower blood and muscle lactate accumulations may, to some extent, act as protective mechanisms: (i) against sickling and thus VOC, that may explain the relatively rare complications and sudden death observed in SCT carriers, and (ii) against the deleterious intracellular effects of lactate and associated acidosis on muscle function, that may explain the elevated presence of SCT carriers among the best short-distance sprinters. 

## Figures and Tables

**Table 1:** Some anthropometric and physiological characteristics and hematological data of the subjects.

	CON (*n* = 15)	SCT (*n* = 15)	*p* Value
Anthropometric and physiological characteristics			
Age (year)	24 (2)	23 (2)	0.052
Body mass (kg)	66 (5)	69 (6)	0.151
P_max_ (W)	210 (170–241)	210 (140–245)	0.475
P_max_ (W·kg^−1^)	3.02 (0.36)	3.01 (0.40)	0.920
DEE (kJ·day^−1^)	10,868 (1474)	11,664 (1321)	0.131
Hemoglobin and hematological data			
HbS (%)	not present	34.3 (3.6)	na
Hct (%)	43.0 (2.7)	43.2 (2.8)	0.826
MCV (fL)	84.71 (5.37)	80.00 (3.87)	0.010
MCH (pg)	27.29 (2.08)	25.93 (1.39)	0.044
MCHC (g·dL^−1^)	32.17 (0.67)	32.33 (0.56)	0.483
RBC (M·µL^−1^)	5.09 (0.42)	5.42 (0.45)	0.053

Values are mean (SD) or median (min-max). P_max_: maximal power; DEE: daily energy expenditure. Hb: hemoglobin; Hct: hematocrit; MCV: mean cell volume; MCH: mean cell hemoglobin; MCHC: mean cell hemoglobin concentration; RBC: red blood cell; WBC: white blood cell; Lymp: lymphocyte; na: not applicable.

**Table 2:** Blood and muscle data.

	CON (*n* = 15)	SCT (*n* = 15)	*p* Value
Blood lactate concentrations			
[lactate]_b_(r) (mmol·L^−1^)	1.356 (0.336)	1.301 (0.323)	0.648
[lactate]_b_(0) (mmol·L^−1^)	8.59 (1.40) [14]	7.08 (1.57)	0.011
Bicarbonate-dependent muscle pH regulation mechanisms			
CAII (a.u.)	1.28 (0.40) [13]	1.43 (0.36) [14]	0.317
CAIII (a.u.)	1.03 (0.34) [13]	1.05 (0.24) [13]	0.857
NBC (a.u.)	3.98 (0.67) [14]	3.95(1.06) [13]	0.928
Sarcolemmal H^+^ transport			
MCT1 (a.u.)	2.14 (0.53) [14]	2.33 (0.61) [13]	0.392
MCT4 (a.u.)	1.18 (1.75–4.81) [14]	2.70 (1.34–5.78) [14]	0.006
Muscle metabolite concentrations			
[lactate]_m_(0) (mmol·kg^−1^ d.m.)	132 (95–201) [13]	113 (83–130)	0.022
[pyruvate]_m_(0) (mmol·kg^−1^ d.m.)	1.85 (0.63–5.76) [13]	2.07 (1.13–3.59)	0.914
[lactate]_m_(0)/[pyruvate]_m_ ratio	60.7 (26.3–225.5) [13]	55.2 (31.5–99.3)	0.440
[ATP]_m_(0) (mmol·kg^−1^ d.m.)	14.4 (9.0–18.3) [13]	12.6 (9.6–21.9)	0.908
[ADP]_m_(0) (mmol·kg^−1^ d.m.)	6.0 (2.60–9.10) [13]	6.70 (3.2–10.3)	0.903
[ATP]_m_/[ADP]_m_(0)	2.73 (1.22–4.38) [13]	1.96 (1.30–4.56)	0.339
LDH isoform proportions			
M-LDH (%)	0.81 (0.71–0.84) [13]	0.81 (0.63–0.88)	0.610
H-LDH (%)	0.19 (0.16–0.29) [13]	0.19 (0.12–0.37)	0.610
β_2_-adrenergic receptors			
β_2_AR (a.u.)	0.87 (0.13–1.60) [14]	0.25 (0.08–1.53) [11]	0.021

Values are mean (SD) or median (min-max). b: blood, m: muscle; (r): rest; (0): exercise completion. CA: carbonic anhydrases; NBC: sodium bicarbonate cotransporter; MCT: monocarboxylate transporter (lactate/H^+^ symporter); a.u.: arbitrary units; ATP: adenosine triphosphate; ADP: adenosine diphosphate; d.m.: dry muscle, M-LDH and H-LDH: proportion of muscle and heart isoforms of lactate dehydrogenase; β_2_AR: β_2_-adrenergic receptor; [n]: number of subjects if different from total group.

**Table 3:** Blood lactate kinetics and glucose/lactate interaction parameters during recovery.

	CON (*n* = 15)	SCT (*n* = 15)	*p* Value
Blood lactate kinetics parameters			
γ_1_ (min^−1^)	0.207 (0.086) [14]	0.227 (0.104)	0.586
γ_2_ (min^−1^)	0.045 (0.011) [14]	0.061 (0.022)	0.020
[lactate]_b_peak (mmol·L^−1^)	12.1 (1.7) [14]	10.4 (1.6)	0.009
Cross-over point of blood glucose and lactate concentrations			
Concentration (mmol·L^−1^)	5.21 (0.64) [14]	4.98 (0.34)	0.242
Time into recovery (min)	36.5 (26.3–66.9) [14]	30.8 (22.0–36.1)	0.012

Values are mean (SD) or median (min-max). A_1_: amplitude of exponential term describing lactate appearance in the blood; γ: velocity constant denoting the lactate exchange ability between the previously active muscle and the blood; A_2_: amplitude of exponential term describing lactate disappearance from the blood; γ: velocity constant denoting the lactate removal ability; peak: peak value observed during recovery; [n]: number of subjects if different from total group.

## Data Availability

Study protocol and data will be available 5 years following article publication to researchers who provide a methodologically sound proposal. Proposals should be directed to laurent.messonnier@univ-smb.fr. To gain access, data requestors will need to sign a data access agreement.

## Supplementary Materials

The following supporting information can be downloaded at: https://www.mdpi.com/article/10.3390/nu14030501/s1, Table S1: Some anthropometric and physiological characteristics and hematological data of the subjects, Table S2: Blood and muscle data, Table S3: Blood lactate kinetics and glucose/lactate interaction parameters during recovery.
