# Supplementary material for: Lower Muscle and Blood Lactate Accumulation in Sickle Cell Trait Carriers in Response to Short High-Intensity Exercise
Source: Nutrients. 2022 Jan 24;14(3):501. doi: 10.3390/nu14030501 (PMC8838817; doi:10.3390/nu14030501)
Supplement: Supplementary file 1 [file nutrients-14-00501-s001.zip › nutrients-1466494-supplementary.pdf]

nutrients

Table S1. Some anthropometric and physiological characteristics and hematological data of the subjects.

	**CON (n = 10)**	**CONα-t (n = 5)**	**SCT (n = 6)**	**SCTα-t (n = 9)**	**HbS**	**α-thal**	**crossed**
Anthropometric and physiological characteristics							
Age (year)	24 ± 1	25 ± 2	23 ±1	22 ± 1	NS	NS	NS
Body mass (kg)	66 ± 2	66 ± 1	68 ± 3	69 ± 2	NS	NS	NS
P_{max} (W)	194 ± 8	207 ± 10	201 ± 17	211 ± 9	NS	NS	NS
P_{max} ($W \cdot kg^{-1}$)	2.97 ± 0.12	3.13 ± 0.16	2.92 ± 0.17	3.07 ± 0.13	NS	NS	NS
DEE ($kJ \cdot day^{-1}$)	10644 ± 491	11316 ± 600	11353 ± 671	11870 ± 372	NS	NS	NS
Hemoglobin and hematological data							
HbS (%)	not present	not present	38.4 ± 0.4	31.6 ± 0.3	na	0.0237	na
Hct (%)	43.4 ± 0.8	42.3 ± 1.2	43.4 ± 1.5	43.1 ± 0.8	NS	NS	NS
MCV (fL)	87.42 ± 1.16	79.30 ± 1.78	83.50 ± 0.93	77.67 ± 0.92	**0.0324**	**< 0.0001**	NS
MCH (pg)	28.32 ± 0.48	25.22 ± 0.61	27.26 ± 0.26	25.03 ± 0.31	NS	**< 0.0001**	NS
MCHC ($g \cdot dL^{-1}$)	32.36 ± 0.22	31.80 ± 0.18	32.65 ± 0.25	32.12 ± 0.15	NS	**0.0195**	NS
RBC ($M \cdot \mu L^{-1}$)	4.97 ± 0.10	5.35 ± 0.24	5.20 ± 0.19	5.56 ± 0.13	NS	**0.0246**	NS

Values are mean ± SEM. P_{max}: maximal power; DEE: daily energy expenditure. Hb: hemoglobin; Hct: hematocrit; MCV: mean cell volume; MCH: mean cell haemoglobin; MCHC: mean cell haemoglobin concentration; RBC: red blood cell; WBC: white blood cell; Lymp: lymphocyte; NS: non-significant, otherwise p value is reported; na: not applicable.

Table S2. Blood and muscle data.

	**CON (n = 10)**	**CONα-t (n = 5)**	**SCT (n = 6)**	**SCTα-t (n = 9)**	**HbS**	**α-thal**	**crossed**
Blood lactate concentrations							
$[lactate]_b(r)$ ($mmol \cdot L^{-1}$)	1.39 ± 0.09	1.13 ± 0.15	1.32 ± 0.1′	1.38 ± 0.12	NS	NS	NS
$[lactate]_b(0)$ ($mmol \cdot L^{-1}$)	8.84 ± 0.51 (9)	8.12 ± 0.48	6.29 ± 0.29	7.61 ± 0.59	**0.0104**	NS	NS
Bicarbonate-dependent muscle pH regulation mechanisms							
CAII (a.u.)	1.25 ± 0.134 (8)	1.32 ± 0.21	1.43 ± 0.18	1.43 ± 0.11 (8)	NS	NS	NS
CAIII (a.u.)	1.09 ± 0.11 (8)	0.93 ± 0.17	1.01 ± 0.09	1.08 ± 0.10 (7)	NS	NS	NS
NBC (a.u.)	3.94 + 0.27 (9)	4.05 ± 0.15	3.89 ± 0.20 (5)	3.98 ± 0.47 (8)	NS	NS	NS
Sarcolemmal H^+ transport							
MCT1 (a.u.)	2.14 ± 0.17 (9)	2.13 ± 0.28	2.25 ± 0.28 (5)	2.38 ± 0.23 (8)	NS	NS	NS
MCT4 (a.u.)	1.68 ± 0.50 (9)	1.11 ± 0.48	2.38 ± 0.28	3.15 ± 0.46 (8)	**0.0092**	NS	NS
Muscle metabolite concentrations							
$[lactate]_m(0)$ ($mmol \cdot kg^{-1}$ d.m.)	133 ± 12 (8)	127 ± 6	111 ± 2	107 ± 6	**0.0218**	NS	NS
$[pyruvate]_m(0)$ ($mmol \cdot kg^{-1}$ d.m.)	2.38 ± 0.48 (8)	1.71 ± 0.50	2.13 ± 0.78	1.88 ± 0.15	NS	NS	NS
$[lactate]_m(0)/[pyruvate]_m$ ratio	72.4 ± 20.6 (8)	101.3 ± 24.5	57.1 ± 6.4	60.4 ± 6.3	NS	NS	NS
$[ATP]_m(0)$ ($mmol \cdot kg^{-1}$ d.m.)	13.34 ± 1.20 (8)	15.22 ± 1.35	15.60 ± 1.97	13.03 ± 1.09	NS	NS	NS
$[ADP]_m(0)$ ($mmol \cdot kg^{-1}$ d.m.)	7.66 ± 1.07 (8)	5.84 ± 0.99	7.32 ± 0.86	6.48 ± 0.68	NS	NS	NS
$[ATP]_m/[ADP]_m(0)$	2.07 ± 0.35 (8)	2.88 ± 0.47	2.30 ± 0.47	2.17 ± 0.25	NS	NS	NS
LDH isoform proportions							
M-LDH (%)	81 ± 2 (8)	80 ± 1	83 ± 2	79 ± 3	NS	NS	NS
H-LDH (%)	19 ± 2 (8)	20 ± 1	17 ± 2	21 ± 3	NS	NS	NS
β_2-adrenergic receptors							
β_2AR (a.u.)	0.85 ± 0.13 (9)	0.87 ± 0.29	0.20 ± 0.03 (5)	0.64 ± 0.21 (6)	**0.0270**	NS	NS

Values are mean ± SEM. b: blood, m: muscle; (r): rest; (0): exercise completion. CA: carbonic anhydrases; NBC: sodium bicarbonate cotransporter; MCT: monocarboxylate transporter (lactate/H^+ symporter); a.u.: arbitrary units; ATP: adenosine triphosphate; ADP: adenosine diphosphate; d.m.: dry muscle, M-LDH and H-LDH: proportion of muscle and heart isoforms of lactate dehydrogenase; β_2AR: β_2-adrenergic receptor; (n): number of subjects if different from total group; NS: non-significant, otherwise p value is reported.

Table S3. Blood lactate kinetics and glucose/lactate interaction parameters during recovery.

	**CON (n = 10)**	**CONα-t (n = 5)**	**SCT (n = 6)**	**SCTα-t (n = 9)**	**HbS**	**α-thal**	**crossed**
Blood lactate kinetics parameters							
A_1 (mmol·L^{-1})	14.9 ± 3.2 (9)	11.3 ± 2.4	10.5 ± 1.7	11.5 ± 2.5	NS	NS	NS
$_1$ (min^{-1})	0.201 ± 0.031 (9)	0.218 ± 0.035	0.227 ± 0.040	0.227 ± 0.038	NS	NS	NS
A_2 (mmol·L^{-1})	-22.1 ± 3.6 (9)	-17.9 ± 2.4	-15.0 ± 1.9	-17.3 ± 2.7	NS	NS	NS
$_2$ (min^{-1})	0.044 ± 0.004 (9)	0.045 ± 0.004	0.069 ± 0.011	0.056 ± 0.006	**0.0168**	NS	NS
[lactate]$_b$peak (mmol·L^{-1})	12.5 ± 0.6 (9)	11.5 ± 0.6	10.2 ± 0.6	10.6 ± 0.5	**0.0185**	NS	NS
Cross-over point of blood glucose and lactate concentrations							
Concentration (mmol·L^{-1})	5.23 ± 0.19 (9)	5.17 ± 0.36	4.97 ± 0.15	4.99 ± 0.12	NS	NS	NS
Time into recovery (min)	40.3 ± 4.3 (9)	35.5 ± 3.6	26.5 ± 1.7	32.0 ± 1.5	**0.0157**	NS	NS

Values are mean ± SEM. A_1: amplitude of exponential term describing lactate appearance in the blood; : velocity constant denoting the lactate exchange ability between the previously active muscle and the blood; A_2: amplitude of exponential term describing lactate disappearance from the blood; : velocity constant denoting the lactate removal ability; peak: peak value observed during recovery; (n): number of subjects if different from total group; NS: non-significant, otherwise p value is reported.
